# Real-Time *In Situ* Spectroscopic and Electrochemical Analysis of Ion–Water–Polymer Interactions at Functionalized PEDOT Interfaces

**DOI:** 10.1021/acs.analchem.4c06327

**Published:** 2025-05-26

**Authors:** Chia-Hsin Lin, Ya-Chen Gong, Hsuan-Yu Chen, Heng-Liang Wu, Shyh-Chyang Luo

**Affiliations:** † Department of Materials Science and Engineering, 33561 National Taiwan University, No. 1, Sec. 4, Roosevelt Road, Taipei 10617, Taiwan; ‡ Center for Condensed Matter Sciences and Center of Atomic Initiative for New Materials, 33561 National Taiwan University, No. 1, Sec. 4, Roosevelt Road, Taipei 10617, Taiwan; § International Graduate Program of Molecular Science and Technology, 33561 National Taiwan University, Taipei 10617, Taiwan

## Abstract

Understanding water dynamics at material interfaces is crucial for applications in biotechnology, electrochemistry, and energy systems. In this study, we employed *in situ* Fourier transform infrared spectroscopy and electrochemical quartz crystal microbalance with dissipation monitoring to investigate the hydration states and ion interactions of functionalized poly­(3,4-ethylenedioxythiophene) (PEDOT) films. By applying controlled potentials, we monitored ion absorption and desorption while using Gaussian fitting to analyze the O–H stretching bands. Our results revealed that sulfate ions (SO_4_^2–^) compete with water molecules at PEDOT interfaces with hydroxyl groups, whereas perchlorate ions (ClO_4_^–^) exhibit minimal interference due to their weak water interactions. For PEDOT functionalized with zwitterionic phosphorylcholine groups, higher levels of intermediate water and nonfreezing water mitigated dehydration in saline environments. This work highlights the synergy between electrochemical and spectroscopic methods for real-time analysis of ion–water–polymer interactions, providing critical insights into interfacial phenomena regulated by applied potential.

## Graphical abstract

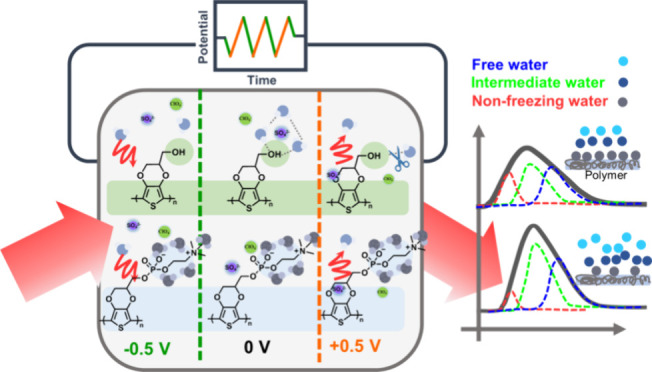

## Introduction

Water is one of the most important and common elements in the biological system and everywhere around us. Although water has a simple chemical structure, a more thorough understanding of H_2_O is still needed. The most stable state, at a temperature below 4 °C, forms a highly ordered tetrahedral structure. On the other hand, the liquid state of water exhibits various configurations, which are associated with other molecules in the environment and the interaction between the material interfaces and surroundings. According to previous studies that Tanaka et al. proposed, from differential scanning calorimetry (DSC) measurement, the hydration state of water inside the polymers can be characterized into three types: nonfreezing water (NFW), intermediate water (IW), and free water (FW).^1,2^ FW stays the farthest from the polymer with a bulk-water-like structure, which is easily affected by the environment outside the materials. However, NFW is strongly connected to the polymers and is hard to freeze even below −100 °C. The characteristic of IW lies between FW and NFW. IW and NFW are also called bound water.^2^ Each type of water has its respective meaning in the biological systems, which has not yet been fully understood,^3^ especially the existence of IW, which can act as a bridge connecting the biomolecules and the materials. The protein fouling to a material has been discussed over the years. The antifouling ability of a material is claimed to be correlated to the hydration structures of water on the material surfaces and the interaction with the proteins. It is well-known and well-developed that the hydrophilic polymers containing an ethylene glycol (EG) unit can bond tightly to water and form a water layer to prevent the initial adhesion of biomolecules through sufficient hydrogen bonding.^4^ Nevertheless, the emergence of zwitterionic polymers, including sulfobetaine-based, carboxybetaine-based, and 2-methacryloyloxyethyl phosphorylcholine (MPC) polymers, has become the dominating materials due to their excellent antifouling ability and the stability in liquid.^5–10^ Notably, phosphorylcholine (PC) groups, inspired by the polar phospholipids on cell membranes, have gained prominence due to their superior antifouling capabilities compared to extended EG chains.^11^ From the previous study, this kind of polymer is inert in the structure of vicinal water.^12^ PC-based polymers cause minimal influence on the surrounding water structure, exhibiting a unique “hydrophobic hydration” phenomenon attributed to intramolecular interactions with PC groups.^13,14^ Several molecular dynamics simulations have discussed that water forms different local structures around this zwitterionic group.^15^ Due to the completely different behavior of water, this makes the issue of water structure on different biomaterials a vital phenomenon that is valuable for in-depth exploration.

The conducting polymer (CP) poly­(3,4-ethylenedioxythiophene) (PEDOT) is a material that is widely used in the field of bioelectronics. In their pristine form, PEDOT thin films offer a versatile platform for various applications, from biosensing to flexible integration with other materials, including hydrogels and inorganic systems. One of the advantages of PEDOT is its versatility in incorporating any functional group, which makes it easy to enhance the bonding with biomolecules to elevate the precision of biosensors. Moreover, covalently bonding the surface with specific targets like antibodies, DNA, and so forth is widely used.^16,17^ PEDOT is a valuable coating material with biocompatibility, stability, and cost-effectiveness. Consequently, investigating the interplay at the interface between PEDOT derivatives and the surrounding environment represents a biotechnology exploration field. The hydrophilic functional group, PC, can also be synthesized on the PEDOT, which makes the coating antifouling and enhances its feasibility in bioelectronics.^18^

The dynamic of water molecules has been explored by nuclear magnetic resonance,^19^ DSC,^20^ and vibrational spectroscopic studies, such as Fourier transform infrared spectroscopy (FTIR), Raman spectroscopy, and sum frequency generation spectroscopy (SFG).^21–25^ The state of water molecules in the polymers has been widely investigated by analyzing the O–H stretching region from IR spectroscopy. Previous studies have revealed that multiple factors contribute to the characteristics of the O–H stretching region, leading to several sub-bands, which may vary by the applied interpretation methods.^26–31^ Determining the key factor that governs the state of water structure within polymers has proven to be challenging and complex, often requiring qualitative calculations for a comprehensive understanding.

Biological systems typically involve a mix of molecules and salts essential for maintaining function.^32–34^ This study focuses on the water absorption behavior under the influence of different salts and applied potentials, which can link to the protein absorption behavior. The series has been widely used to elaborate the phenomena in the biological and physicochemical fields. Specific effects of these ions on water structure have been classified into “structure-making (kosmotropic ion)” and “structure-breaking (chaotropic ion)”.^35–37^ We chose sodium ion as the control cation. The order of sodium salt can be listed as follows: NaSCN> NaClO_4_ > NaI > NaNO_3_ ≈ NaBr > NaCl > pure water ≈ NaF ≈ Na_2_SO_4_.^38^ We monitored the state of water influenced by the kosmotropic and chaotropic anion in PEDOTs with different functional groups by using IR spectroscopy. The applied potential to a surface will be associated with anions and cause the rearrangement of the water molecules’ configuration. Upon potential sweeps, we can control the movement of the salt near the polymer interface. In the present study, electrical quartz crystal microbalance with dissipation (EQCM-D) was applied as a tool to monitor the ion absorption/desorption behavior on the surface of PEDOT derivative films, and IR absorption spectroscopy was used to acquire the spectrum of water at the interface. This study aims to unravel the complex interplay among water structure, polymer functional groups, and ionic environments. Understanding the distribution and dynamics of FW, IW, and NFW at biointerfaces is essential, as these water types influence biomolecular absorption. By investigating how functional groups, electric potentials, and salts collectively shape water structure, we strive to lay the foundation for the precise control of biomolecular interactions on polymer surfaces. Ultimately, achieving accurate measurements and interpretations of water dynamics will be pivotal in designing next-generation biointerfaces with tailored antifouling properties and enhanced biosensing capabilities.

## Experimental Section/Methods

### Materials

The monomer EDOT and tetrabutylammonium perchlorate (TBAP) were purchased from Tokyo Chemical Industry Co., Ltd. EDOT-OH was purchased from Sigma-Aldrich (USA). Three salts used for the electrolytes, sodium sulfate, sodium chloride, and sodium perchlorate, were purchased from Honeywell International Inc. Lithium perchlorate and acetonitrile were obtained from Alfa Aesar. Dodecyl sulfate sodium salt and dioctyl sulfosuccinate sodium salt were purchased from ACROS. EDOT-PC was from Dr. Hsiao-hua Yu, Academia Sinica, and the synthesizing method followed previously published procedures.^60^ Two monomers, EDOT-EG_3_OH and EDOT-EG_3_OMe, were synthesized using the process that was published before.^4^ All of the above chemicals were used without further purification.

### Preparation of PEDOT Derivative Films

The monomer EDOT and EDOT-OH aqueous solutions were prepared by dissolving 10 mM EDOT-OH, 50 mM dodecyl sulfate sodium, and 100 mM LiClO_4_ in DI water. On the other hand, EDOT-PC, EDOT-EG_3_OH, and EDOT-EG_3_OMe were dissolved in nonaqueous acetonitrile with 10 mM of each monomer, 50 mM dioctyl sulfosuccinate sodium salt, and 100 mM TBAP. The electropolymerization process was performed using an Autolab PGSTAT101 potentiostat (Metrohm, The Netherlands) in a three-electrode setup with an *in situ* FTIR cell. The condition of electropolymerization was set identically for both aqueous and nonaqueous phases. In the aqueous phase, the electrolyte of the reference electrode was composed of Ag/AgCl (saturated KCl), and the Ag/Ag^+^ electrode with 100 mM TBAP in acetonitrile was used in the nonaqueous phase. The CV scan was conducted for the electrodeposition process with one cycle from −0.6 to 1.1 V (V vs Ag/AgCl or Ag/Ag^+^) at a scan rate of 100 mV s^–1^ under room temperature.

### AFM Characterization of Film Thickness

The film thicknesses of the PEDOT derivatives were characterized using a Bruker JPK NanoWizard 3 atomic force microscope operated in tapping mode, employing a Bruker ScanAsyst-Air silicon nitride probe. For each sample, a small scratch was carefully introduced with a sharp blade to expose the underlying substrate, thereby allowing us to determine the thickness of the film. AFM height images were acquired across the scratched region, and the film thickness was calculated as the height difference between the exposed substrate and the top surface of the surrounding film. To ensure the reliability and reproducibility of the measurements, this procedure was performed at a minimum of three different locations on each film, and the average height difference was reported as the film thickness.

### EQCM-D Measurements

EQCM-D expands the applications of normal QCM-D by integrating the bias conditions. By configuration of a three-electrode system and application of an external potential to the surface, the setup allows EQCM-D to modulate surface potentials. We utilized the QEM 401 Q-Sense electrochemical module (Biolin Scientific, Västra Frölunda, Sweden) integrated with an Autolab PGSTAT128N potentiostat (Metrohm, The Netherlands) for electrochemical experiments. These experiments employed a three-electrode setup with a Ag/AgCl reference electrode containing 3.4 M KCl. Voltage modulation followed the fixed process. The applied voltage started with the OCP and was conducted in 0.1 V decrements from the OCP to – 0.5 V and then swept back from – 0.5 to 0.5 V. Finally, the applied potential gradually went back to 0 V, which completed one cycle. Each potential was held for 3 min.

### Water Contact Angle Measurements

The contact angles were measured by using a contact angle goniometer (Sindatek, Taiwan). All measurements were conducted at room temperature (approximately 25 °C) by depositing a 3 μL water droplet onto the gold surfaces and the various PEDOT derivative films. On each sample, a minimum of five measurements were performed at different locations to ensure the reproducibility of the results, and the average contact angle values are displayed in Figure 1.

**Figure 1:**
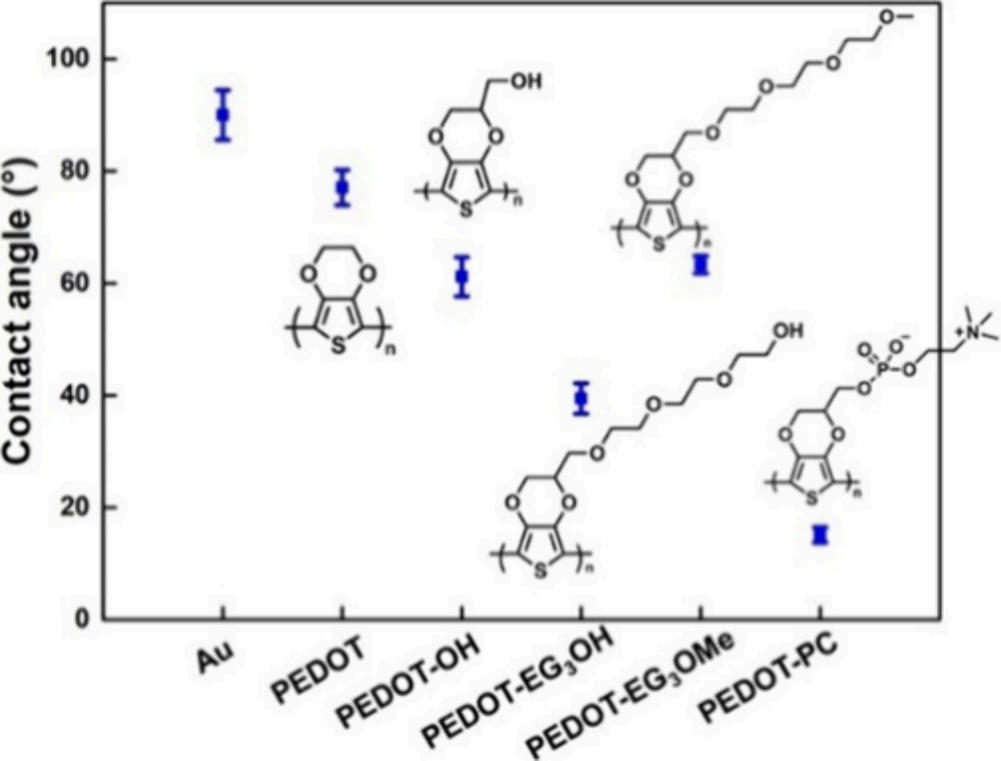
Contact angle results of pristine gold, PEDOT, PEDOT-OH, PEDOT-EG_3_OH, PEDOT-EG_3_OMe, and PEDOT-PC.

### DSC Measurements

The hydrated polymer samples were fully immersed in DI water for over 2 weeks. After they were removed from the water and the surface moisture was washed off, about 3 mg of each sample was placed in aluminum pans and sealed. The sealed samples were kept at room temperature overnight before the DSC measurements to achieve a stable state. During the analysis, the samples were cooled to −65 °C at a rate of 3.0 °C/min, held at that temperature for 10 min, and then heated to 50 °C at the same rate under a nitrogen flow (20.0 mL min^–1^). Following the DSC measurements, the pans were pierced, and the samples were dried in a vacuum oven at 100 °C to determine the dry polymer weight. The quantitative analysis of the weight of the water content (including IW, FW, and NFW) was based on the results of other previous studies.

### *In Situ* FTIR and Spectroscopic Analysis

A thin Au film was prepared on a Si prism using electroless deposition.^61^ The spectroelectrochemical cell was assembled by fixing a glass cell and the prism with a circular O-ring in between. The Au-coated prism was a working electrode, and the other two electrodes were immersed in the electrolytes. Millipore deionized water with a resistivity of 18.2 MΩ was used to prepare the electrolytes. Counter and reference electrodes were a platinum foil and Ag/AgCl electrodes. After the PEDOT derivative film deposition, this spectroelectrochemical cell was set into a Thermo Nicolet 6700 FTIR spectrometer equipped with a HgCdTe (MCT) detector.^43,62^ The spectral resolution of the *in situ* FTIR spectra was 4 cm^–1^. The acquisition time was ∼10 s for each spectrum. Three electrolytes, including 100 mM Na_2_SO_4_, NaCl, and NaClO_4_, were used based on the Hofmeister series. The potential was applied by an Autolab PGSTAT101 potentiostat (Metrohm, The Netherlands). The background spectrum was collected at the OCP. The applied potential was swept from the OCP to – 0.5 V, from −0.5 to +0.5 V, and then back to 0 V.

## Results and Discussion

### Surface Characteristics and Water Content of PEDOT Derivatives

The water contact angle (WCA) primarily revealed the wettability of PEDOT derivatives with functional groups. We characterized the pristine PEDOT derivative films in air after electropolymerization, as shown in Figure 1. The WCA of the deposited Au film is around 85°. The WCA of PEDOT without any functionalization is about 78° and gradually drops with one OH functionalizing PEDOT-OH and then three EG functionalizing PEDOT-EG_3_OH. In contrast, the WCA rises when the terminal OH group changes to a methoxy group (PEDOT-EG_3_OMe). The PEDOT-PC behaves the most hydrophilic due to the simultaneous existence of the negatively charged phosphate bonded to a small and positively charged choline group on the side chain. The hydrophilicity of long EG chains, resulting from the polymer’s multihydrogen bonding sites, is less efficient than localized cation and anion. The hydrophobic hydration concept of MPC has also been investigated by SFG spectroscopy.^15^ The water configuration showed that the hydrogen-up and hydrogen-down water coexisted near anionic phosphate and cationic choline sites instead of the specific and static orientation of hydrogen bonding on OH groups. This resulted in ultrafast hydrogen-bond fluctuation at the zwitterionic interface.^39^ This contributed to the apparent differences in their hydrophilicity.

From the DSC measurement in Figure 2, the FW in the PEDOT crystallizes at around −16 °C, forms a huge and sharp peak for full crystallization, and melts again beyond 0 °C during the heating process. After removing the FW of each polymer, we observed the cold crystallization of IW below −20 °C with a broad and noncontinuous exothermic reaction and a broad melting peak starting at about −20 °C. On the other hand, NFW is noncrystallizable, which would not present on the DCS thermogram. The water encapsulated within the functionalized PEDOT derivatives exhibits distinctions from the FW owing to higher interaction between water molecules and materials.

**Figure 2:**
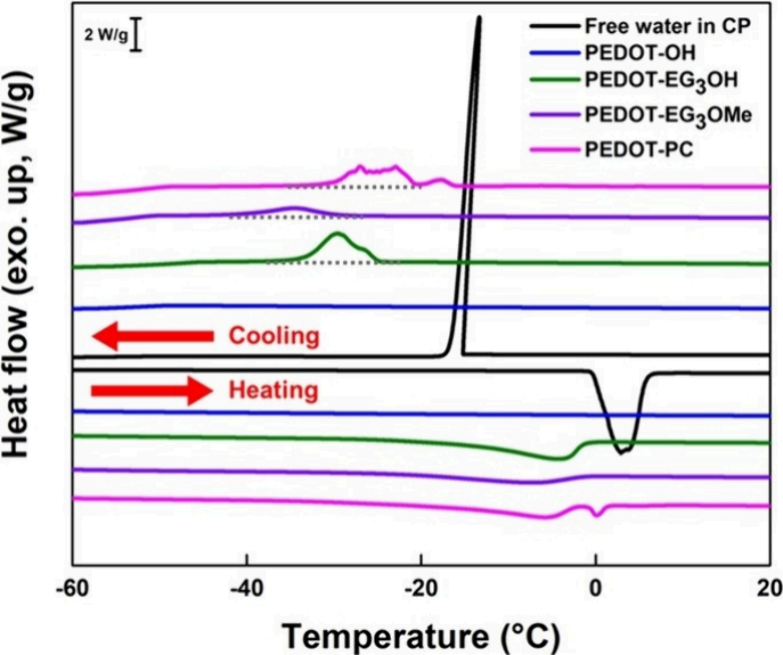
DSC thermograms of pure water in CP (black), PEDOT-OH (blue), PEDOT-EG_3_OH (olive), PEDOT-EG_3_OMe (purple), and PEDOT-PC (pink) during the heating and cooling scan.

We calculated the hydrated water contents of the IW and NFW that occupied per mole of PEDOT derivatives, as listed in Table 1. The method followed the approach described in a previous study.^4^ *W*_total_ represents the total amount of water occupying per mole of PEDOT derivatives. After subtracting the values of FW and IW, we can obtain the amount of NFW. This shows that the *W*_NFW_ in PEDOT-PC is much higher than in PEDOT-EG_3_OH and PEDOT-EG_3_OMe. It is worth noting that PC can promote the formation of water clusters.^40^ This can explain why the *W*_total_, including the FW content, is especially high within PEDOT-PC. The DSC result of PEDOT-OH presents no peak due to its low hydration state, which is hard to calculate. This further illustrates that the water configuration of zwitterionic materials is distinct from others. Combining the WCA and DSC results, PC groups can strongly bond with water molecules and hold more water inside this material, giving rise to their excellent hydrophilicity and antifouling ability

**Table 1:** Hydrated Water Contents in Each PEDOT Derivative (mol mol^–1^) of PEDOT-OH, PEDOT-PC, PEDOT-EG_3_OH, and PEDOT-EG_3_OMe^a^

mol mol^–1^	PEDOT-OH	PEDOT-PC	PEDOT-EG_3_OH	PEDOT-EG_3_OMe
*W*_total_		15.37	6.19	5.44
*W*_IW_		2.00	1.82	1.03
*W*_FW_		2.01	0.35	0.50
*W*_NFW_		11.36	4.02	3.91

a *W*_total_: total water content in per mole of PEDOT derivatives; *W*_IW_: IW content in per mole of PEDOT derivatives; *W*_FW_: FW content in per mole of PEDOT derivatives; and *W*_NFW_: NFW content in per mole of PEDOT derivatives.

### Spectroelectrochemical Characterization of PEDOT

FTIR spectroscopy is a powerful and sensitive method to characterize materials’ composition, molecular structure, and chemical environment.^41–43^ The conformation of PEDOT in a doping state has been well-studied by *ex situ* methods to date.^44–48^ PEDOT can be electrochemically synthesized in various solvents, such as aqueous, nonaqueous, and ionic liquids. With *in situ* FTIR spectroscopy, we can thoroughly observe the peak evolution from the neutral form of PEDOT to the oxidation state.^49^ Electropolymerization was performed in a three-electrode setup with an *in situ* FTIR cell (Figure 3a). The neutral state of PEDOT is at −0.9 V, which contains the most neutral species.^46^ In this study, the PEDOT film was prepared by scanning the potential from −0.6 to 1.1 V. After electropolymerization, the film on Au-coated Si prism was directly immersed in the solution with Na_2_SO_4_, NaCl, or NaClO_4_ in the *in situ* FTIR system, and the anions were chosen based on the Hofmeister series (Figure 3b). The representative FTIR peaks are given in Table 2. With the potential applied from the open circuit potential (OCP) to −0.5 V, the downward peak at ∼1536 cm^–1^ and the upward peak b at 1468 cm^–1^ are obtained. On the other hand, when the applied voltage is above +0.1 V, the upward peak located at 1549 cm^–1^ starts to appear (Figure 3c). The peaks a and b at 1468 and 1549 cm^–1^ are the most apparent vibrational transitions of the asymmetric C=C stretching mode of the thiophene ring. The structure of this PEDOT transition can be denoted by a benzoid state at a reduced potential and a quinoid state at an oxidized potential. The structures are shown in Figure 3d. The peak assigned to the quinoid state is formed at a positive potential. Peak b associated with the benzoid state appears at the negative potential. Peak c at 1434–1424 cm^–1^ is assigned to the symmetric C=C stretching of PEDOT with quinoid and benzoid states. The peaks from 1200 to 1300 cm^–1^ (peak d) are the assignments of doping-induced bands resulting from changes in the conjugated backbone of the thiophene ring during oxidation.^50^ The results indicate that the state of pristine PEDOT chains is not identical, and the segments of quinoid and benzoid states transform, corresponding to the applied potential. With the *in situ* FTIR spectra, we can clearly observe the reversible structure transition during the stepwise and continuous potential sweep of CPs.

**Table 2:** Table of Infrared Band Assignments and Comparison of the Absorption Bands of PEDOT during Structure Transition

wavenumber (cm^–1^)	assignments	refs
1630–1640	δ(HOH)	^22^
1549 (peak a)	ν(C=C_asym_) quinoid state	^44,46,51^
1468 (peak b)	ν(C=C_asym_) benzoid state	^44,46,51^
1424–1436 (peak c)	ν(C=C_sym_) PEDOT	^52,51^
1200–1300 (peak d)	ν(C–O–C) + doping induced	^44,45,49,51^

**Figure 3:**
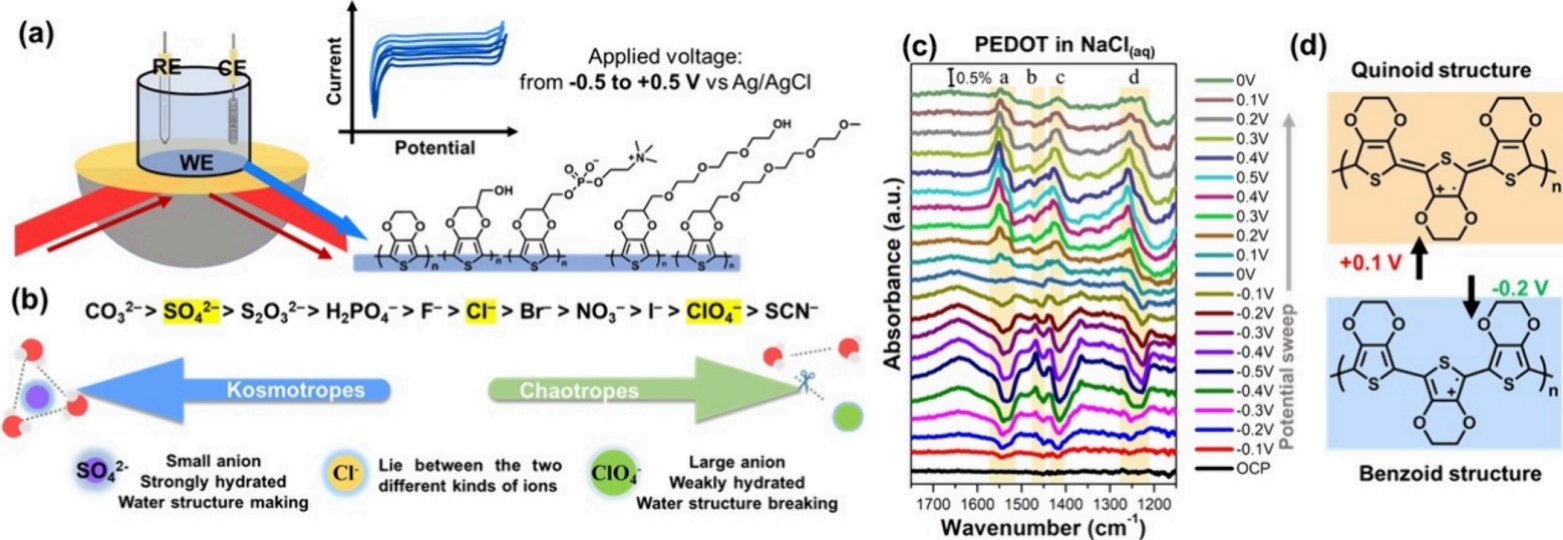
Experimental setup and cell design to carry out *in situ* FTIR measurements. (a) Different functionalized PEDOT derivatives were chosen as materials to monitor the PEDOT derivative/electrolyte interface. The applied potential was swept from OCP to −0.5 V, −0.5 to +0.5 V, and then back to 0 V. (b) The Hofmeister series ranks the anions from kosmotropes to chaotropes. Sulfate, chloride, and perchlorate anions were used in the present study. (c) *In situ* FTIR spectra of PEDOT in 100 mM NaCl. (d) The schematic presentation of the PEDOT transforming between benzoid structure and quinoid structure reversibly.

### Ion Absorption on the PEDOT Derivative Films and Sub-band Variation of O–H Stretching with Potentials in Electrolytes

*In situ* FTIR and EQCM-D provide complementary real-time information crucial for understanding ion–water–polymer interactions within functionalized PEDOT films under various electrolytes and potentials. *In situ* FTIR identifies chemical bonding and functional group change, with the O–H stretching analysis distinguishing free, intermediate, and nonfreezing water states. Complementing the structural view from FTIR, EQCM-D intuitively tracks film-state changes by directly monitoring mass variations from ion and water movement. The observed frequency shifts (Δ*f*) offer semiquantitative insights into the transport processes within the film. Its dissipation change (Δ*D*) further reflects hydration states and interaction dynamics. This combined information assists in constructing models for ion and water absorption/desorption behavior for different PEDOT derivatives under various potentials.

We then tested the ion–water absorption and desorption behaviors in different electrolytes with a potential sweep by EQCM-D. Figure 4a,b displays the real-time EQCM-D responses of PEDOT-PC films under varying applied potentials in Na_2_SO_4_ and NaClO_4_, respectively. Before measurement, the film was equilibrated in the electrolyte followed by a continuous potential sweep from negative to positive. Interestingly, as shown in Figure 4a, applying negative potentials (blue region) resulted in simultaneous increases in both frequency and dissipation. The frequency shift (Δ*f*) reached a maximum of +52.85 Hz, while the dissipation change (Δ*D*) concurrently increased to a maximum of +20.50 × 10^–6^. This behavior arises from two distinct processes induced by negative potential application. First, electrostatic repulsion pushes hydrated SO_4_^2–^ ions out of the PEDOT-PC film, leading to a positive frequency shift. Second, ion departure creates internal voids that, along with PEDOT-PC’s hydrophilicity, allow free water to enter. This results in a more hydrated film and increased energy dissipation. Although water enters the film, the net frequency increases because EQCM-D detects the coupled mass. The strongly coupled hydrated sulfate ions contribute more to the frequency decrease than the incoming free water, making ion expulsion the main cause of the frequency shift. As the potential sweeps from negative to positive (orange region), both frequency and dissipation decrease, indicating anion reabsorption and expulsion of free water that slightly collapses the film and lowers dissipation. However, only about half of the released anions return, indicating an irreversible process. A similar pattern appears in Figure 4b, where negative potential increases frequency and dissipation to +74.47 Hz and +11.89 × 10^–6^ followed by a decrease under positive potential. Compared with unfunctionalized PEDOT (Figure S1), anions struggled to detach at negative potentials due to the positively charged backbone, while increased ion uptake at positive potentials led to a decrease in frequency.

**Figure 4:**
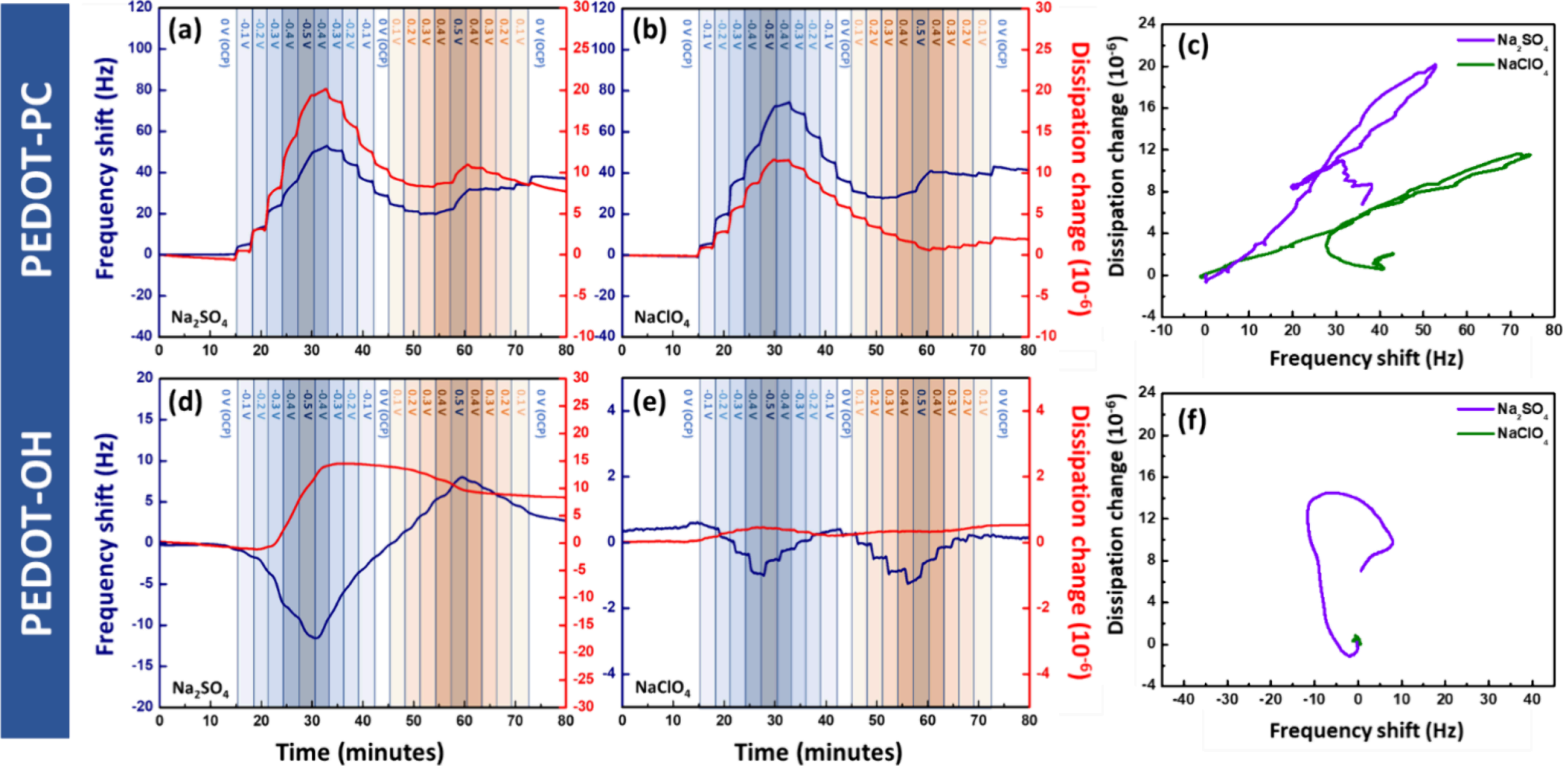
Monitoring the frequency and dissipation shift versus time from −0.5 to +0.5 V using EQCM-D in deionized water with salts. The potential was held for 3 min at every 0.1 V (vs Ag/AgCl). The ion–water absorption of PEDOT-PC in Na_2_SO_4_ and NaClO_4_ is shown in panels a and b, and their Δ*D*–Δ*f* curves are shown in panel c. The ion–water absorption of PEDOT-OH in Na_2_SO_4_ and NaClO_4_ is shown in panels d and e, and their Δ*D*–Δ*f* curves are shown in panel f. The concentrations of NaClO_4_ and Na_2_SO_4_ were 100 mM.

To better compare the structural responses, Figure 4a,b was demonstrated as Δ*D*–Δ*f* curves in Figure 4c. During the initial negative potential phase, both Na_2_SO_4_ (purple line) and NaClO_4_ (green line) systems show positive slopes, indicating that mass loss is accompanied by an increased level of dissipation. The steeper slope in Na_2_SO_4_ suggests greater structural reorganization per unit mass loss. This is due to the strongly hydrated SO_4_^2–^ ions removing more intermediate water as they exit, disrupting the structured hydration layer. The voids within the film allow more free water to enter, resulting in a more disordered, hydrated internal structure and increasing dissipation. In contrast, ClO_4_^–^ ions, being weakly hydrated and chaotropic, induce less disruption to the film’s water structure upon leaving and result in a smaller dissipation change. As a result, the kosmotropic sulfate ion, with its smaller radius and higher hydration capacity, introduces more water into the PEDOT film compared with the weakly hydrated, chaotropic perchlorate ion.

Figure 4d,e shows the real-time EQCM-D responses of PEDOT-OH films under varying potentials in Na_2_SO_4_ and NaClO_4_. PEDOT-OH has polar hydroxyl groups that form hydrogen bonds with water, but compared with the highly hydrated zwitterionic PEDOT-PC, it retains less water in the film at equilibrium. As a result, hydrated Na^+^ ions play a more prominent role. In Figure 4d, negative potentials cause a frequency drop (≈−11.56 Hz) and increased dissipation, indicating that the electrostatically attracted Na^+^ and accompanying free water outweigh the expulsion of hydrated SO_4_^2–^ ions. This mass in the film increases, and water uptake results in a film with a higher water content. Under positive potentials, the frequency rises (≈+8.01 Hz) as Na^+^ and water are expelled, causing a mass loss. In contrast, due to the weak interaction of chaotropic ClO_4_^–^ ions with the film, ion exchange and water rearrangement are largely suppressed. Consequently, frequency changes remain very small across the potential range (≈−1.01 Hz under negative potential, ≈−1.25 Hz under positive potential), and the dissipation change stays close to zero in Figure 4e, indicating that the ion and water layers are not significantly affected by the applied potentials.

Figure 4f presents the Δ*D*–Δ*f* plots derived from Figure 4d,e. For PEDOT-OH in Na_2_SO_4_ (purple line), a steeper negative slope is observed under negative potentials. This is attributed to the limited initial ion movement and a smaller frequency shift. In contrast, the PEDOT-OH film in NaClO_4_ (green line) shows minimal changes in both Δ*D* and Δ*f*, further confirming that the water structure within the film remains unaffected and independent of the applied potential in the weakly hydrated, chaotropic ClO_4_^–^ system.

Compared to EQCM-D, which detects behaviors occurring on the surface of materials within the range of 1 Å to 1 μm, *in situ* FTIR detects changes occurring within the entire region of the PEDOT derivative films, where the PEDOT derivatives interact with the electrolyte and structural or hydration changes. To provide context for the FTIR analysis, the thicknesses of the PEDOT derivative films were measured using atomic force microscopy (AFM) as detailed in the Experimental Section/Methods. Each film was measured at least three times, and the average thickness and standard deviation for each are presented in Table 3.

**Table 3:** Average Thickness and Standard Deviation for PEDOT, PEDOT-OH, and PEDOT-PC Films

PEDOT derivative	average thickness (nm)	standard deviation (nm)
PEDOT	57.3	1.65
PEDOT-OH	32.7	2.29
PEDOT-PC	65.1	10.7

The ion absorption was again confirmed when a potential was applied to the PEDOT derivatives, as depicted in Figure S2. Within the O–H stretching region from 3000 to 3650 cm^–1^, we observed a broad spectral feature indicative of hydrogen bond network formation among water molecules. A series of infrared spectra of PEDOT, PEDOT-OH, and PEDOT-PC were obtained in three different electrolytes, as shown in Figures S3–S5. It is worth noting that the O–H stretching band of water is primarily composed of a broad peak between 3400 and 3200 cm^–1^ in the infrared spectrum.^53^ Morita et al. presented that the intensity of pure water in the *in situ* FTIR system was observed to be 3 times higher with strong absorbance at 3200 cm^–1^.^27^ Importantly, there was a significant difference in the spectrum feature of the bulk water contacting the film surface between the adsorbed water within the polymer film in an attenuated total reflection FTIR (ATR-IR) system. Our investigation showed a noticeable absence or weak presence of O–H stretching bands corresponding to bulk water on a pristine Au-covered prism after water balancing (Figure S6). Consequently, we disregarded the influence of bulk water, and the spectrum changes were based on the potential-dependent water molecule reorientation toward the polymer films in our subsequent discussions. The variations in the spectra result from the water absorption–desorption process occurring under the applied potential. This region exhibits multiple sub-bands, typically comprising three to four components based on hydrogen bond coordination numbers. Initially, we observed a shoulder at the lowest wavenumber around 3200 cm^–1^, characterized by water molecules favoring tetrahedral bonding with each other and limited interaction with the interface material—a phenomenon referred to as “ice-like water”.^54^ Subsequently, the band at approximately 3400 cm^–1^ was assigned to the presence of three-coordinated water structures or small water clusters.^26^ Then, at higher frequencies, around 3540 and 3610 cm^–1^, we observed spectral features related to dimeric and monomeric water molecules, which exhibited a stronger affinity toward the polymer matrix.^31^ These vibrational modes were also found to be potential-dependent in the presence of HClO_4_ and H_2_SO_4_ on the ordered Au (111) electrode due to the reorientation of water molecules.^55^ The potential change not only influenced the salt but also disturbed the relative populations of the orientations of the water molecules. The adsorbed chaotropic anion disrupted the hydrogen bonding within the ice-like structure, rearranging the water molecules at the interface. As a result, we considered two factors in this study: salts and applied potential. Sodium salts were selected for this study because Na^+^ is strategically positioned within the Hofmeister series to minimize the interference with the behavior of anions. To quantify the adsorbed water on PEDOT, we integrated the area of the O–H stretching peaks, as depicted in Figure 5, with detailed data processing provided in Supporting Information Section 1.

**Figure 5:**
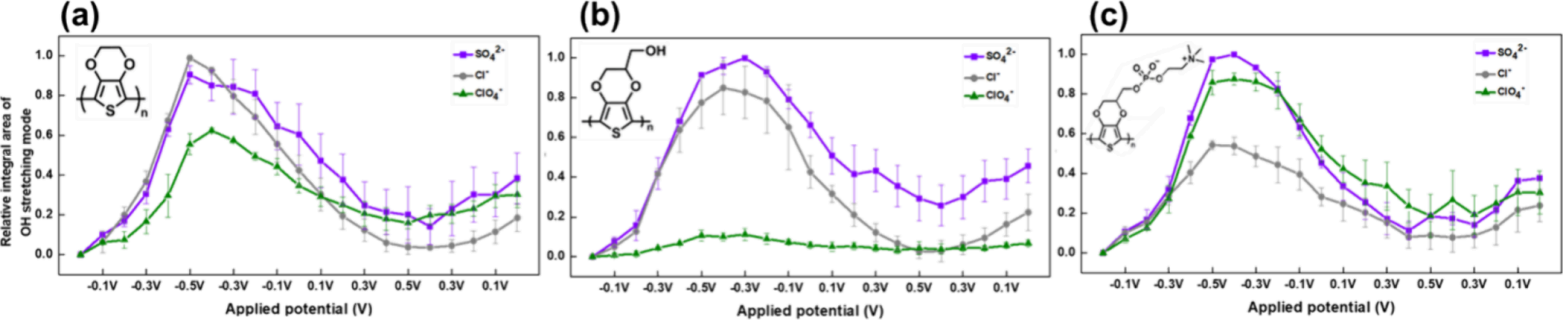
Potential-dependent integrated FTIR peak area of the O–H stretching modes in (a) PEDOT, (b) PEDOT-OH, and (c) PEDOT-PC. The electrolytes are 100 mM Na_2_SO_4_, NaCl, and NaClO_4_.

In Figure 5a, we observed an increase in the level of adsorbed water as the potential swept from the OCP to – 0.5 V on PEDOT without functionalization. This behavior can be attributed to the reduced concentration of cations/anions at the interface, increasing adsorbed water within the PEDOT derivatives. Our results confirmed that SO_4_^2–^, a strongly hydrated kosmotropic anion, led to greater water absorption in PEDOT than the weakly hydrated, chaotropic ClO_4_^–^. The central peak corresponding to the O–H stretching of PEDOT in the Na_2_SO_4_ solution was observed at 3460 cm^–1^, whereas it appeared at 3434 cm^–1^ in NaCl and 3426 cm^–1^ in NaClO_4_ at negative potentials (Figure S3). This discrepancy indicates that the states of water molecules vary in solution depending on the presence of salt. When the cations/anions leave the surface of the polymer, it causes fluctuations in water molecules. In the solution containing sulfate ions, they build a strong bond between water molecules. When we apply a negative potential, the water molecules near the interface tend to form stronger bonds with the polymer.

On the other hand, weakly hydrated perchlorate ions do not compete with the water molecules around the polymer interface under OCP. Low-frequency water is present in the solution containing perchlorate ions. As a result, the red-shift phenomenon follows the Hofmeister series from the left-hand to the right-hand side. For PEDOT-OH, which possesses sufficient OH moieties, removing SO_4_^2–^ led to a pronounced increase in water absorption over −0.2 V, as shown in Figure 5b. Even upon the reabsorption of the SO_4_^2–^ ions, an apparent water absorption peak at positive potentials remained. This suggested that the water absorption behavior was irreversible on the PEDOT derivative films. The central FTIR peak corresponding to the O–H stretching mode of PEDOT-OH was observed at 3467 cm^–1^ in the Na_2_SO_4_ solution and shifted to 3438 cm^–1^ in the NaCl solution at negative potentials (Figure S4).

In contrast, in the NaClO_4_ solution, we observed low-intensity signals across the entire spectrum, and the results were presented independent of the applied potential (Figure S4c). Water molecules bonded to the OH group were not significantly affected by the weakly hydrated ClO_4_^–^ ions, which also corresponded to previous EQCM results. Historically, the Hofmeister series has been elucidated based on ion–water interactions, which can result in the “salt in” or “salt out” effect on proteins. However, the ion–macromolecule–water interaction is also a crucial factor that may strongly influence the behavior of water molecules.^35^ Many different types of antifouling materials have been developed. Materials with EG units can bond tightly to water and form a water layer, which gives rise to their hydrophilicity, to prevent the initial adhesion of biomolecules. The result further verified that sufficient OH moiety groups could resist ion disturbances when the potential is applied. As for PEDOT-PC, characterized by a zwitterionic side chain, although the total integration showed a lower water content at the negative potential in NaCl, the trends of the spectra in the three salts were similar (Figure 6c). The series of spectra are shown in Figure S5. Compared with the other two PEDOT derivatives, the variation of the position of central peaks corresponding to the O–H stretching is more stable for PEDOT-PC: the highest FITR peak positions were observed at 3474, 3467, and 3461 cm^–1^ in Na_2_SO_4_, NaCl, and NaClO_4_ solutions at negative potentials, respectively. Compared to PEDOT and PEDOT-OH, PEDOT-PC shifted toward higher wavenumbers in the presence of all salts, indicating stronger interactions between the adsorbed water and the polymer. This observation aligned with previous research proposing that adding salts did not diminish the hydration of PEDOT-PC owing to the unique structural characteristics of water molecules surrounding PC groups.^56^

**Figure 6:**
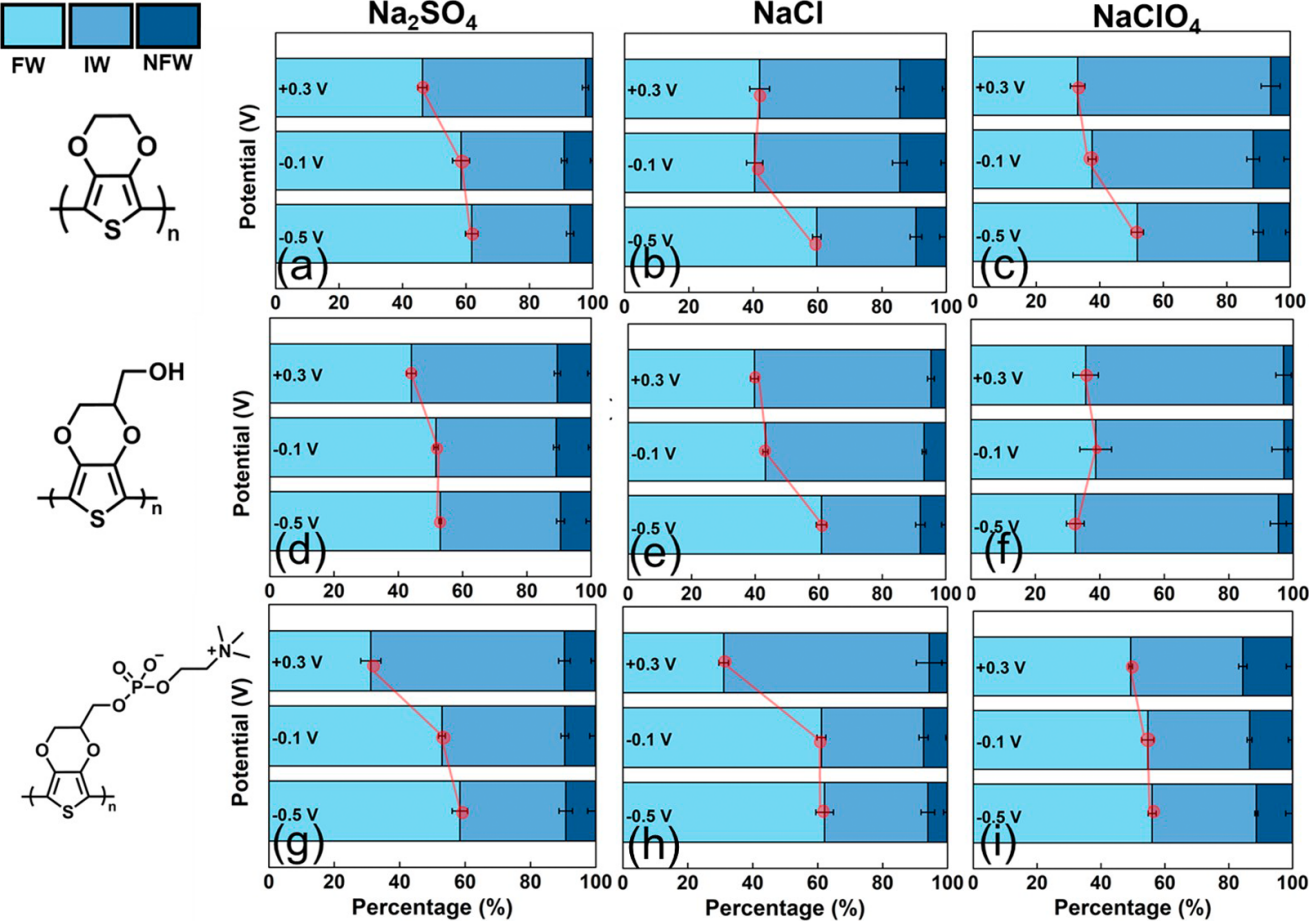
Stacked chart of the percentage of the FW, IW, and NFW with their respective relative areas at PEDOT in (a) Na_2_SO_4_, (b) NaCl, (c) NaClO_4_; PEDOT-OH in (d) Na_2_SO_4_, (e) NaCl, (f) NaClO_4_; and PEDOT-PC in (g) Na_2_SO_4_, (h) NaCl, and (i) NaClO_4_.

In addition to the distribution of the structured water molecules, we analyzed the O–H stretching modes with a Gaussian function. The O–H bond oscillator strength of water molecules is related to the number of donated hydrogen bonds.^55,57,58^ The evolution of peaks of the sub-bands will vary with the methods, measurements, and materials. In the present study, we adopted the peaks at 3295, 3460, and 3590 cm^–1^ as three components according to a previous study.^26,54^ Brubach et al. measured the temperature evolution of liquid water and proposed that the three Gaussian components were assigned to three dominating populations of water molecules.^26^ They suggested that the lowest frequency at Gaussian 3295 cm^–1^ was assigned to molecules in the tetrahedral water structure, which is close to the bulk water and denoted as FW. Then, the 3460 cm^–1^ was associated with water molecules that have an average degree of connection with materials between FW and NFW. This type of molecule was referred to as IW. The third, 3590 cm^–1^, was ascribed to water molecules being poorly connected to their environment (other water molecules) due to their stronger affinity to materials. Morita et al. have also proposed that the peak around 3600 cm^–1^ comprised two peaks, 3628 and 3558 cm^–1^, followed by a broad and mild shoulder in the 3400–3200 cm^–1^ region.^27^ This main peak can be denoted by NFW. As a result, these three peaks are assigned as the characteristic peaks of FW, IW, and NFW at 3295, 3460, and 3590 cm^–1^ in our study.^27^ The value of *R* ^2^ of each Gaussian fitting result here is higher than 0.99. The results are shown in Figures S7–S9 for PEDOT, PEDOT-OH, and PEDOT-PC. The potentials of – 0.5, – 0.1, and +0.3 V were selected to illustrate the distribution change of water in different electrolytes. To investigate the water structure variation with potentials, the red, green, and blue dashed lines represent the decomposition into three Gaussians at −0.5, −0.1, and +0.3 V with their relative areas. From Figures S3 to S5, the intensities of water absorption nearly disappeared at higher potentials. As a result, the spectral signal obtained at +0.5 V is very weak, and the fitting data will have errors. We took fitting curves of +0.3 V rather than +0.5 V to avoid misleading results. The stacked charts demonstrate the composition of the three variables in the relative value of each state of water molecules (Figure 6).

As mentioned before, ClO_4_^–^ acts as a water structure breaker, which has been extensively discussed. First, for PEDOT without any functionalization, the composition of FW decreased when applied to high potentials and dropped to below 50% for SO_4_^2–^ and Cl^–^ (Figure 6a,b). As the surface salt concentration increases and with Na^+^ ions being insensitive to potential, the most easily perturbed “free water” within the polymer film tends to migrate outward to balance the concentration with the external environment. ClO_4_^–^ causes the evaluation of dimer or trimer of the water molecules around PEDOT, so the FW percentages even fell below 40% at −0.1 V (Figure 6c). On the other hand, the salt-induced dehydration phenomenon also happened on PEDOT-OH (Figure 6d,e). Among them, the most remarkable result is that FW in PEDOT-OH with potential was irregular in ClO_4_^–^ due to the low peak intensity in the series, which made the fitting result hard to identify (Figure 6f). The FW percentages also dropped apparently on PEDOT-PC at high potentials; however, the phenomenon happened in SO_4_^2–^ and Cl^–^ (Figure 6g,h). In a previous study, Morita et al. demonstrated that freezing water in an MPC-based copolymer is easily balanced with the biological surface without perturbing the NFW in the polymer at a high concentration of saline solution.^59^ Poly­(2-methoxyethyl acrylate) was considered a nonbiocompatible polymer containing less IW and NFW. The absorbance of ATR-IR spectra for poly­(2-methoxyethyl acrylate) drastically decreased due to the dehydration in 0.15 M NaCl solution. However, the results of the MPC-based copolymer only found a peak shift from 3200 to 3500 cm^–1^. They suggested that the FW was exchanged with the NaCl solution that contacted the material surface to prevent the dehydration of the polymer. In Figure 6g,h, the content of FW was both 31% in PEDOT-PC at 0.3 V. The content of bound water mainly remained in the PEDOT derivative films. This finding, consistent with the observations of Morita et al., supports the effective retention of intermediate water (IW) in PEDOT-PC under positive potential. By contrast, although the ClO_4_^–^ still caused the dehydration of the polymer during the potential sweep, the salt did not alter the state of water of PEDOT-PC (Figure 6i). The shape of the O–H stretching remains steady during the whole potential. It is said that water molecules in the hydration layer act as very efficient lubricants to get an antifouling ability. Zhang et al. have demonstrated that the salts slightly altered the intra- and intermolecular electrostatic repulsion from a nanotribological view; however, the hydration state remained unaffected by the salt concentration in the PMPC brush system.^56^ They also found that SO_4_^2–^ affected the hydrogen bonding network around the PC groups, which lowered the lubricity in the PMPC brush system. In correspondence with our investigation, the FW contents showed a lower value in contact with Na_2_SO_4_ due to the kosmotropic effect. In Figure 7, we summarize the movement of water during the potential sweep of three PEDOT derivatives based on EQCM-D and *in situ* FTIR data through illustrations and propose four points:

1. The kosmotropic SO_4_^2–^ causes a more significant hydration effect on PEDOT derivatives than chaotropic ClO_4_^–^.
2. The dehydration happens at positive potentials when the anions get close to the PEDOT derivatives. However, the weakly hydrated and water-breaking ClO_4_^–^ cannot affect the water that bonds to the PEDOT-OH; as a result, hydration and dehydration are suppressed.
3. The peaks are static on PEDOT–PC. The presence of kosmotropic ions appears to facilitate the retention of intermediate water (IW) on PEDOT–PC.
4. By changing the applied potential and the salt types, the O–H stretching components can be well-fitting and analyzed by Gaussian fitting.

**Figure 7:**
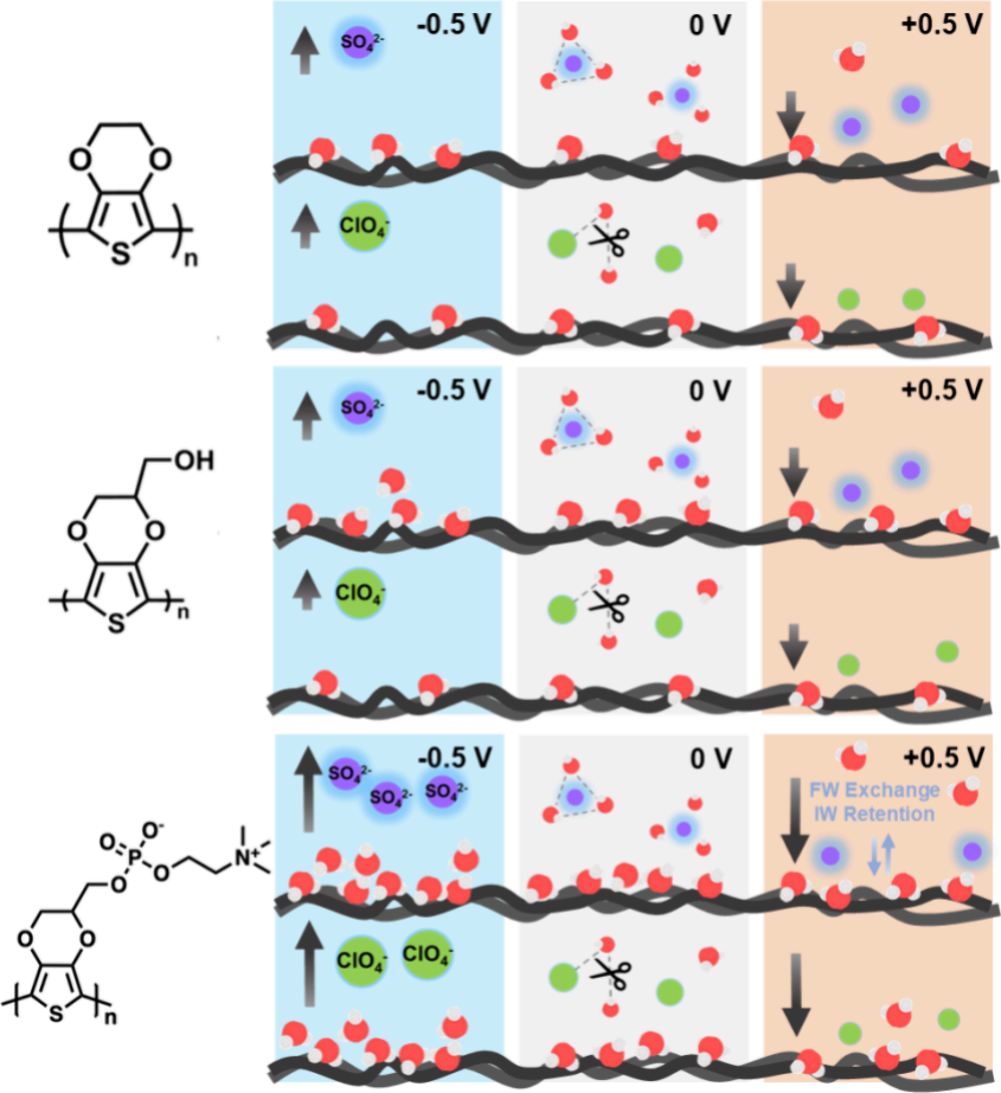
Schematic illustrations of the movement of water molecules on the PEDOT derivatives. The arrow in the diagram indicates the direction in which ions move with changes in the voltage.

To further confirm the sluggish interaction of ClO_4_^–^ with the OH groups, we experimented with PEDOT-EG_3_OH and PEDOT-EG_3_OMe in 100 mM NaClO_4_. The results are shown in Figure S10. The EG chains provide more hydrogen bonding sides for water molecules, which show an apparent decrease in WCA. However, the intensity of the series of PEDOT-EG_3_OH was not obvious (Figure S10a), the same as the result of PEDOT-OH. The peaks shift to a larger wavenumber at 3525 cm^–1^, indicating that the water molecules that adsorbed on the PEDOT-EG_3_OH have a stronger affinity than PEDOT-OH. After substituting the OH end groups to the methoxy group, the appearance of FW indicated that the ClO_4_^–^ can cause hydration/dehydration to PEDOT-EG_3_OMe. This result further confirms that when OH groups are present, perchlorate ions cannot cause fluctuations in water molecules.

In addition, to address the potential spectral interference from the ν­(O–H) modes of the pristine PEDOT-OH and PEDOT-EG_3_OH films in the analysis of water absorption and desorption, we conducted ATR-FTIR measurements on pristine PEDOT, PEDOT-OH, and PEDOT-EG_3_OH films before exposure to aqueous media (Figure S11a). The corresponding ν­(O–H) modes are shown in Figure S11b, where PEDOT-OH exhibits the most prominent O–H peak at ∼3275 cm^–1^. To examine its potential influence on the O–H peaks, we compared FTIR spectra of PEDOT-OH films at −0.5 V in Na_2_SO_4_, NaCl, and NaClO_4_ with the FTIR result of the pristine PEDOT-OH film in air (Figure S12a). The O–H peaks in Na_2_SO_4_ and NaCl were distinct and stronger than the peak of the pristine PEDOT-OH film (∼3275 cm^–1^). These results suggest the following: (1) The intensity of the O–H peaks of the pristine PEDOT-OH film in air is relatively weak compared to the intensity of water absorption bands in the Na_2_SO_4_ and NaCl solutions. (2) The prominent O–H peaks of the PEDOT-OH film differ from the characteristic peaks of IW and NFW (3460 and 3590 cm^–1^ in our study), thus having minimal effect on water-state quantification. While the low OH intensity of the pristine PEDOT-OH film in air might exhibit a minor effect on the FW component at 3295 cm^–1^, it does not alter the overall conclusions and observed trends in our analysis. Similarly, for PEDOT-EG_3_OH, the pristine film before exposure to aqueous media showed a weak O–H peak at ∼3248 cm^–1^, while the PEDOT-EG_3_OH film at −0.5 V in NaClO_4_ displayed a peak at 3525 cm^–1^ (Figure S12b). The low intensity and spectral separation confirm that polymer-derived O–H signals do not significantly interfere with the water absorption/desorption analysis in our experiments.

## Conclusions

This study utilized *in situ* techniques, including FTIR and EQCM-D, to uncover the dynamic interactions of water, ions, and functionalized conducting polymers. *In situ* FTIR revealed ion absorption/desorption dynamics and water structure transformations under potential control, with Gaussian fitting highlighting the hydration effects of the SO_4_^2–^ and ClO_4_^–^ ions. EQCM-D further provided real-time insights into ion movement and polymer hydration, emphasizing the protective role of PC groups.

Building on previous research, we confirmed that protein absorption, cell adhesion, and growth are closely linked to intermediate water (IW), which is found exclusively in biocompatible polymers. Additionally, materials with antifouling properties exhibit a high proportion of nonfreezing water (NFW), which hinders biomolecule adhesion and can disrupt normal biological functioning. The distinct roles of free water (FW), IW, and NFW have significant implications for biological systems, and a deeper understanding of these hydration states could greatly advance biosensing technologies and their applications. Expanding on these insights, our study provides critical advancements in antifouling strategies for implantable bioelectrodes. While PEG and zwitterionic polymers have been widely explored for antifouling applications, their interactions with surface potential remain insufficiently understood, particularly in electrode systems. Changes in surface potential not only influence the ionic strength of the surrounding solution but also impact antifouling efficacy, further complicated by electrostatic effects on protein absorption. Given this complexity, our integration of *in situ* FTIR and electrochemical QCM-D represents a significant step forward in elucidating these interactions. By providing a more comprehensive analysis of how hydration layers and antifouling characteristics respond to surface charge variations, our approach enables meaningful comparisons with other antifouling materials and contributes to the development of biofouling-resistant surfaces for implantable bioelectrode applications.

## Supplementary Material



## Notes

Additional experimental details for EQCM-D and infrared spectroscopy, including the infrared spectra of PEDOT derivatives, peak fitting results for the O–H stretching region, and EQCM-D measurements of ion absorption on PEDOT in deionized water with salts (PDF)
