# Supplementary material for: Real-Time In Situ Spectroscopic and Electrochemical Analysis of Ion–Water–Polymer Interactions at Functionalized PEDOT Interfaces
Source: Anal Chem. 2025 May 26;97(22):11402–12. doi: 10.1021/acs.analchem.4c06327 (PMC12163894; doi:10.1021/acs.analchem.4c06327)
Supplement: Supplementary file 1 [file ac4c06327_si_001.pdf]

Supporting Information

Real-Time In Situ Spectroscopic and Electrochemical Analysis of Ion-Water-Polymer Interactions at Functionalized PEDOT Interfaces

Chia-Hsin Lin,[†] Ya-Chen Gong,[†] Hsuan-Yu Chen,[‡,§] Heng-Liang Wu,[,‡,§] and Shyh-Chyang Luo,[*,†,§]*

[†]Department of Materials Science and Engineering, National Taiwan University, No. 1, Sec. 4, Roosevelt Road, Taipei 10617, Taiwan

[‡]Center for Condensed Matter Sciences and Center of Atomic Initiative for New Materials, National Taiwan University, No. 1, Sec. 4, Roosevelt Road, Taipei 10617, Taiwan

[§]International Graduate Program of Molecular Science and Technology, National Taiwan University, Taipei 10617, Taiwan

*Heng-Liang Wu, Email: hengliangwu@ntu.edu.tw

*Shyh-Chyang Luo, Email: shyhchyang@ntu.edu.tw,

Supplementary Text

Section S1: Quantitative analysis of adsorbed water in PEDOT films via FTIR integration and normalization.

Figure S1: Monitoring the frequency shift versus time from −0.5 V to +0.5 V using EQCM-D in deionized water with salts.

Figure S2: Ion absorption peaks on CP during potential applied.

Figure S3: Series of infrared spectra of PEDOT in three different salts.

Figure S4: Series of infrared spectra of PEDOT-OH in three different salts.

Figure S5: Series of infrared spectra of PEDOT-PC in three different salts.

Figure S6: A spectrum of pure Au film in DI water with 100 mM NaCl.

Figure S7: The peak fitting results of PEDOT at O−H stretching region in three electrolytes using three Gaussian components.

Figure S8: The peak fitting results of PEDOT-OH at O−H stretching region in three electrolytes using three Gaussian components.

Figure S9: The peak fitting results of PEDOT-PC at O−H stretching region in three electrolytes using three Gaussian components.

Figure S10: The series of *in-situ* FTIR spectra of (a) PEDOT-OH, (b) PEDOT-EG_3OH, and (b) PEDOT-EG_3OMe in 100 mM $NaClO_4$ at each potential. The applied potential was swept from OCP to –0.5 V, –0.5 V to +0.5 V, and then back to 0 V (vs. Ag/AgCl).

Figure S11: A spectrum of pristine PEDOT, PEDOT-OH, and PEDOT-EG_3OH films before aqueous media exposure and the corresponding ν (O–H) region.

Figure S12: Comparison of ν (O–H) spectra for pristine and hydrated PEDOT-OH and PEDOT-EG_3OH films under –0.5 V in 100 mM electrolyte solutions.

Section S1:

Quantitative Analysis of Adsorbed Water in PEDOT Films via FTIR Integration and Normalization

To quantify the adsorbed water content in PEDOT derivative films, we analyzed the *in-situ* FTIR spectra by integrating the O–H stretching region (approximately 3000–3650 cm^{-1}). To ensure comparability across samples, the integrated areas were normalized to account for minor variations in film thickness. For each electrolyte condition (Na_2SO_4, NaCl, and $NaClO_4$), the O–H region was integrated across the full range of applied potentials. For each PEDOT derivative (PEDOT, PEDOT-OH, and PEDOT-PC), the maximum integrated O–H area observed among all electrolyte and potential conditions was designated as the reference value (normalized to 1). All other values for the same film type were then scaled relative to this maximum. This normalization approach enables a consistent comparison of relative water uptake across different electrolytes and potentials. The resulting normalized O–H integrated areas are shown in **Figure 5**, highlighting the variations in water adsorption behavior across the three electrolytes for each PEDOT derivative.

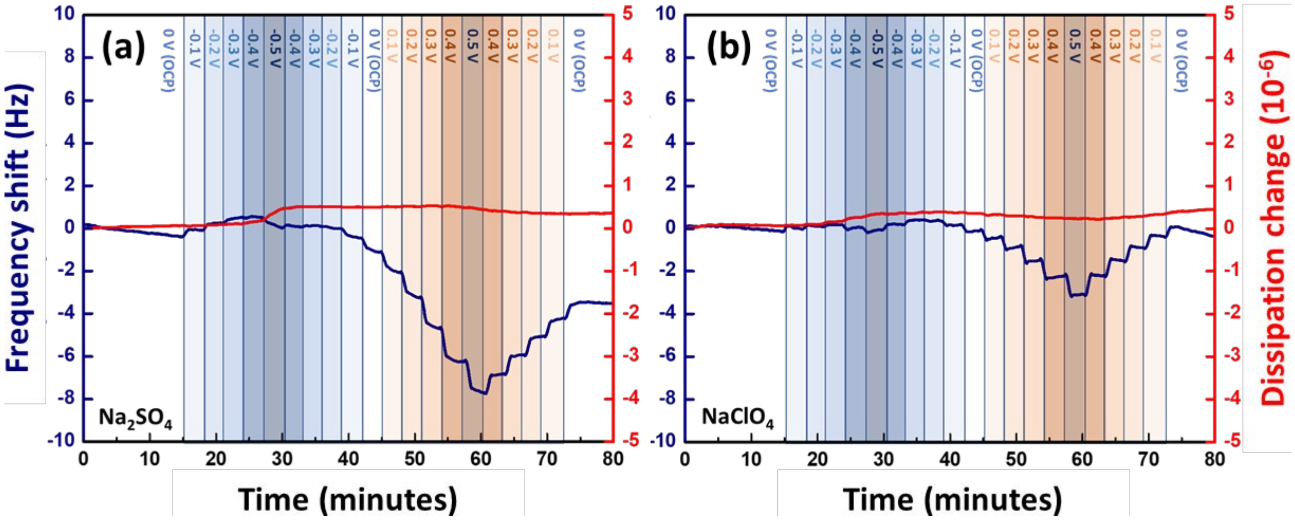

Figure S1. Monitoring the frequency and dissipation shift versus time from −0.5 V to +0.5 V using EQCM-D in deionized water with salts. The potential was held at every 0.1 V (vs. Ag/AgCl) for three minutes. The ion-water absorption of PEDOT in Na_2SO_4 and $NaClO_4$ are shown in (a) and (b). The concentration of $NaClO_4$ and Na_2SO_4 was 100 mM.

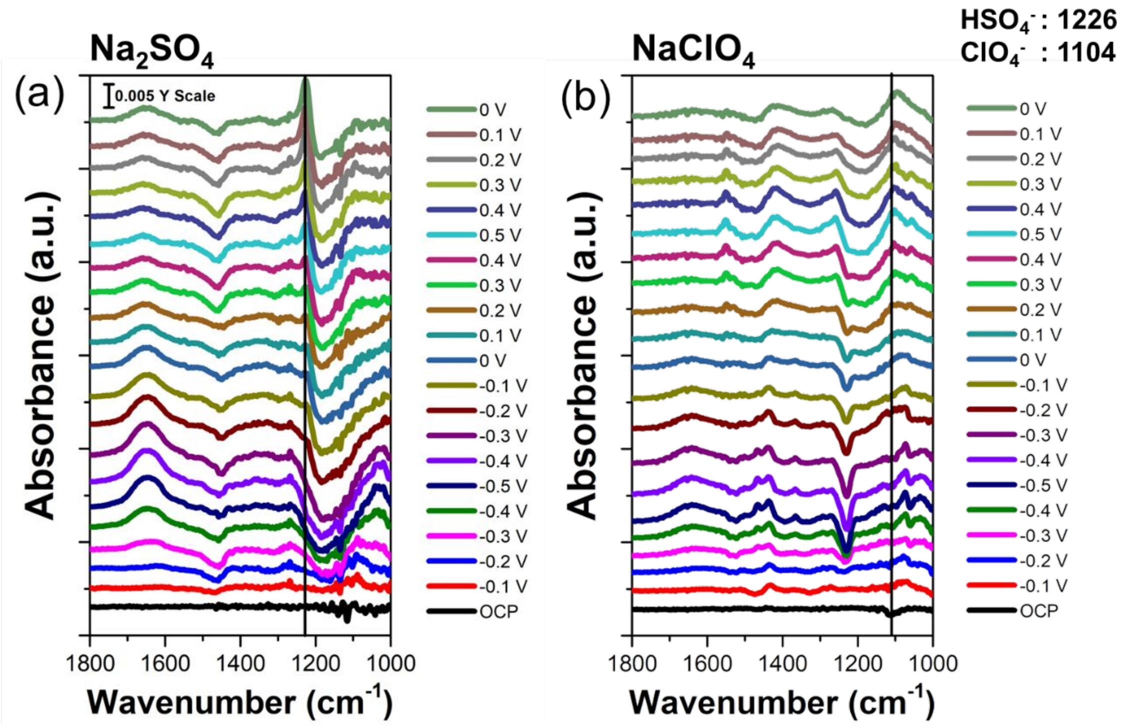

Figure S2. Ion absorption peaks on CP during the potential applied. SO_4^{2-} (a) and ClO_4^- (b) absorption on CP during a potential sweep from OCP to –0.5 V, –0.5 V to +0.5 V, and finally back to 0 V (vs. Ag/AgCl) at a rate of 2 mV s^{-1}.[1]

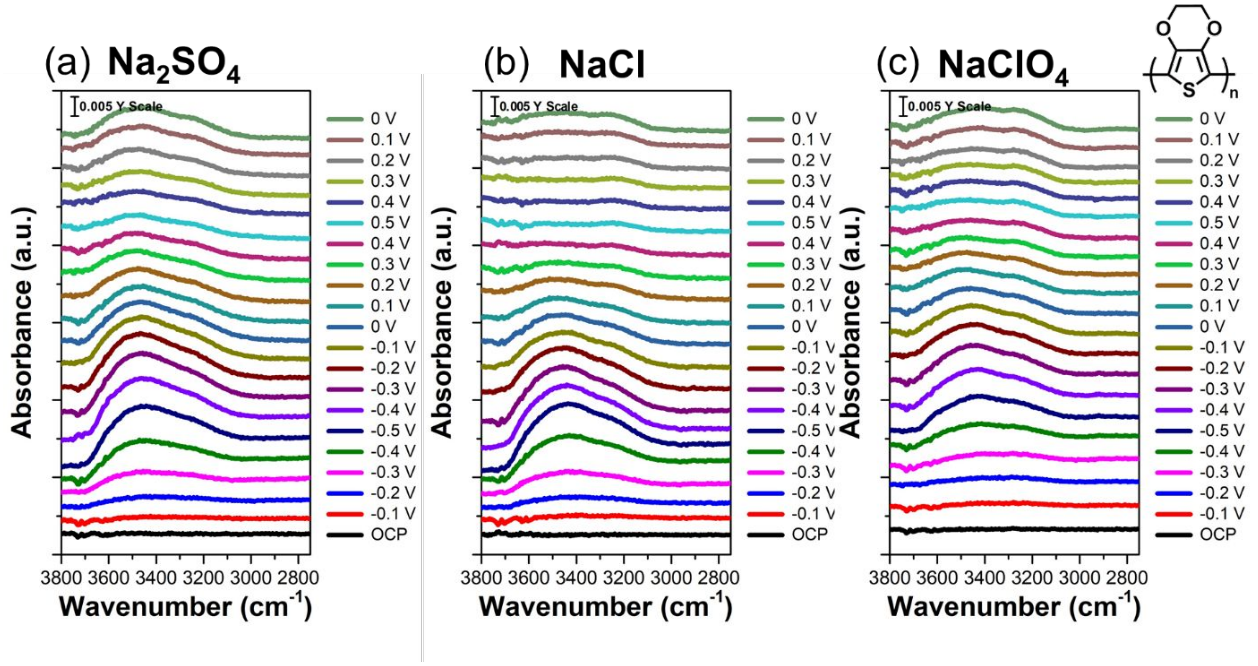

Figure S3. Series of infrared spectra of PEDOT in (a) 100 mM Na_2SO_4, (b) 100 mM NaCl, and (c) 100 mM $NaClO_4$ during the potential sweep from OCP to –0.5 V, –0.5 V to +0.5 V, and then back to 0 V (vs. Ag/AgCl) with a scan rate of 2 mV s^{-1}.

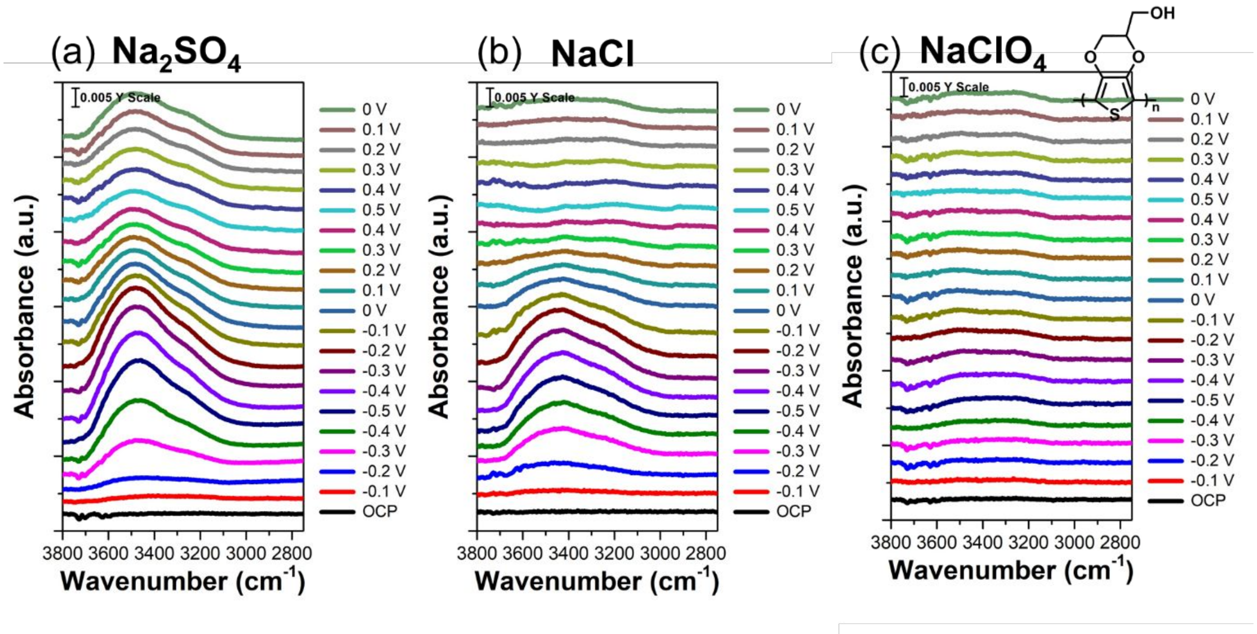

Figure S4. Series of infrared spectra of PEDOT-OH in (a) 100 mM Na_2SO_4, (b) 100 mM NaCl, and (c) 100 mM $NaClO_4$ during the potential sweep from OCP to –0.5 V, –0.5 V to +0.5 V, and then back to 0 V (vs. Ag/AgCl) with a scan rate of 2 mV s^{-1}.

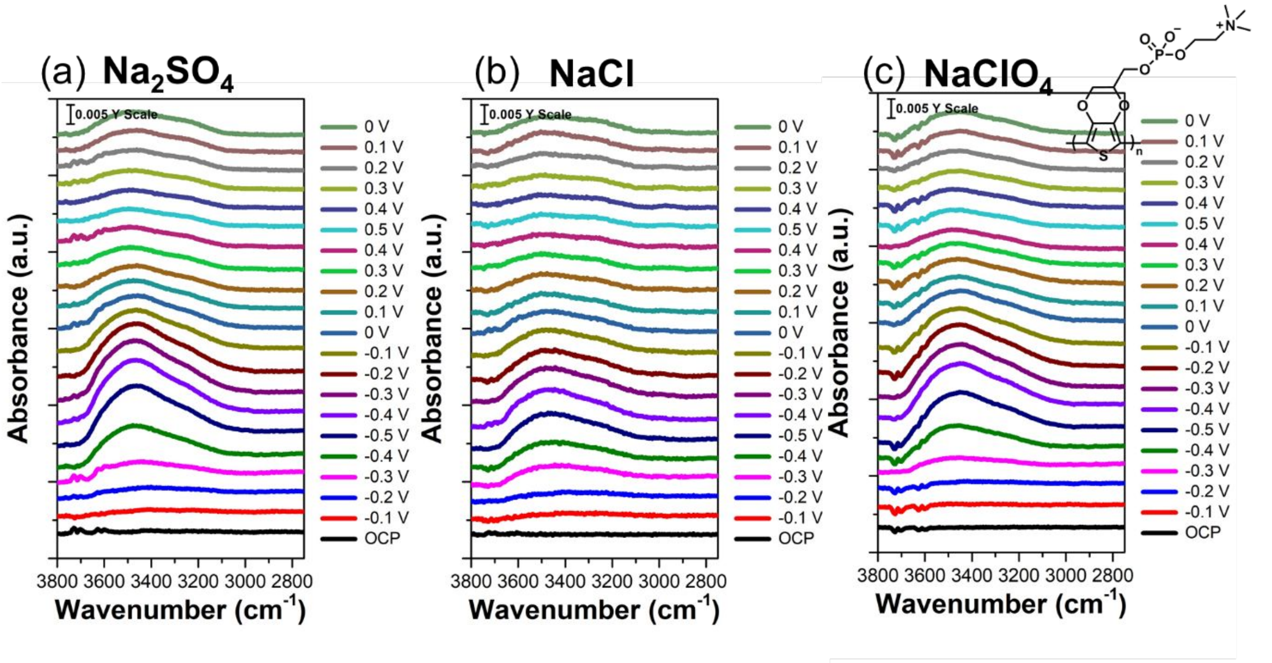

Figure S5. Series of infrared spectra of PEDOT-PC in (a) 100 mM Na_2SO_4, (b) 100 mM NaCl, and (c) 100 mM $NaClO_4$ during the potential sweep from OCP to –0.5 V, –0.5 V to +0.5 V, and then back to 0 V (vs. Ag/AgCl) with a scan rate of 2 mV s^{-1}.

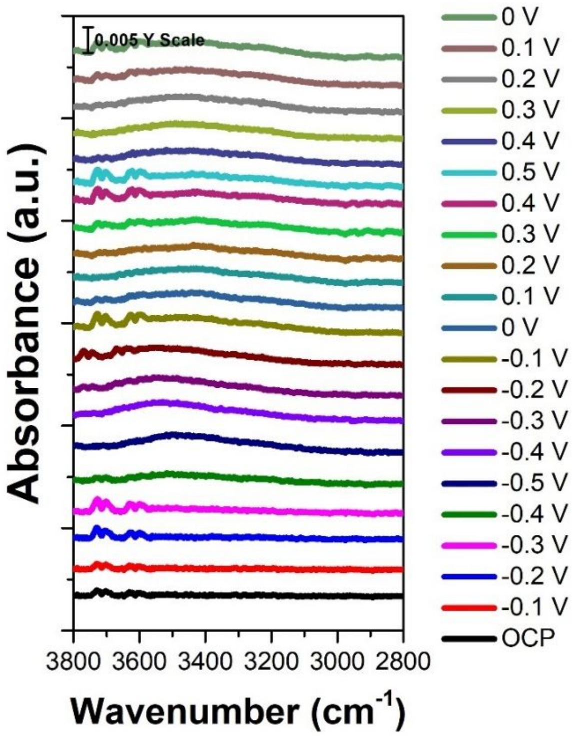

Figure S6. A spectrum of pure Au film in DI water with 100 mM $NaClO_4$. The series shows no intensity of O–H stretching band from bulk water.

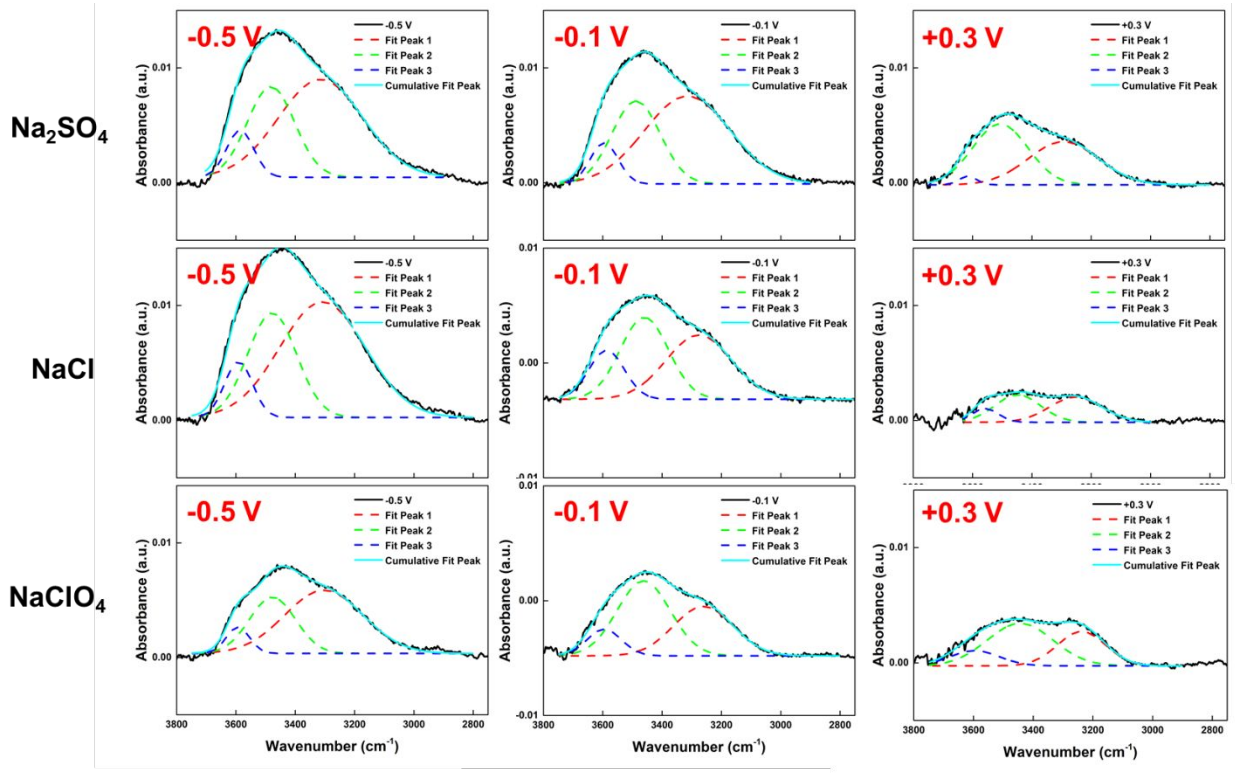

Figure S7. The peak fitting results of PEDOT at OH stretching region in three electrolytes by using three Gaussian components: 3295, 3560, and 3590 cm^{-1}, corresponding to free water (FW), intermediate water (IW), and non-freezing water (NFW), respectively. –0.5 V, –0.1 V, and +0.3 V are chosen for three potentials to present the distribution change of the water in different electrolytes.

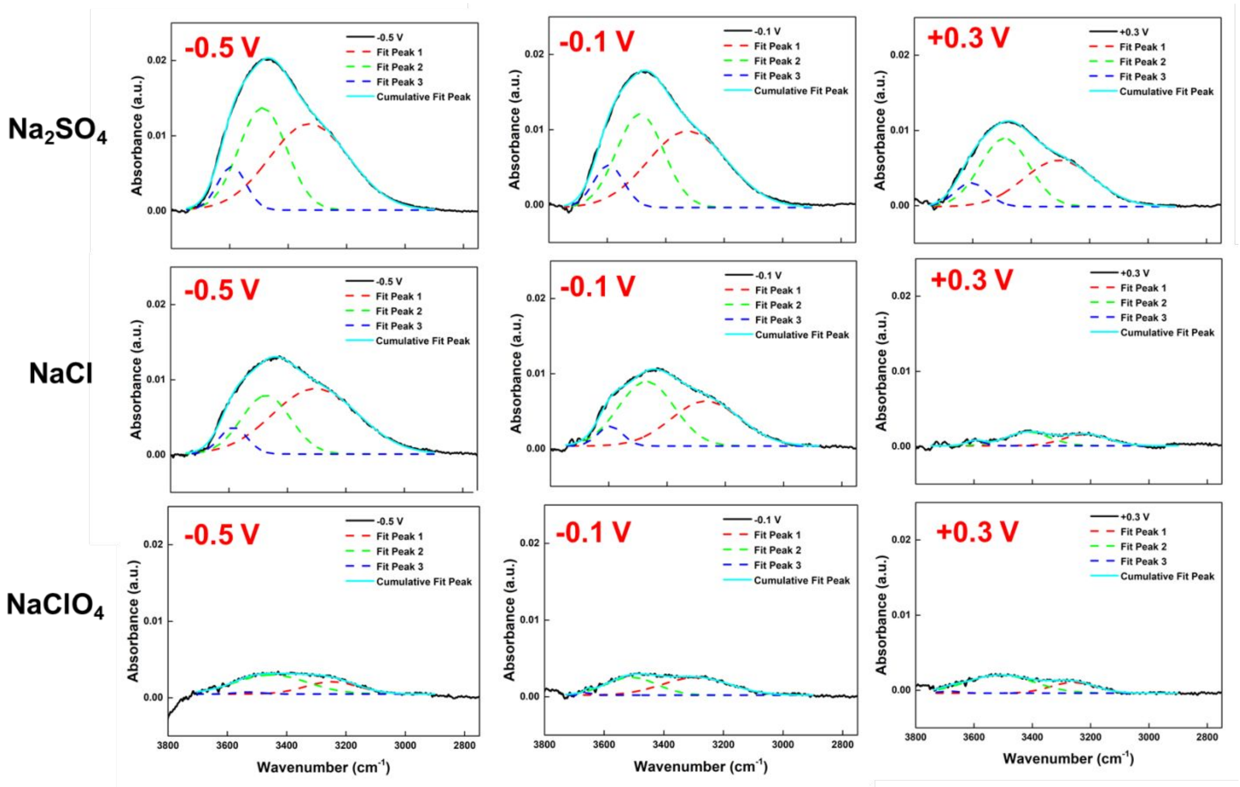

Figure S8. The peak fitting results of PEDOT-OH at OH stretching region in three electrolytes by using three Gaussian components: 3295, 3560, and 3590 cm^{-1}, corresponding to FW, IW, and NFW, respectively. –0.5 V, –0.1 V, and +0.3 V are chosen for three potentials to present the distribution change of the water in different electrolytes.

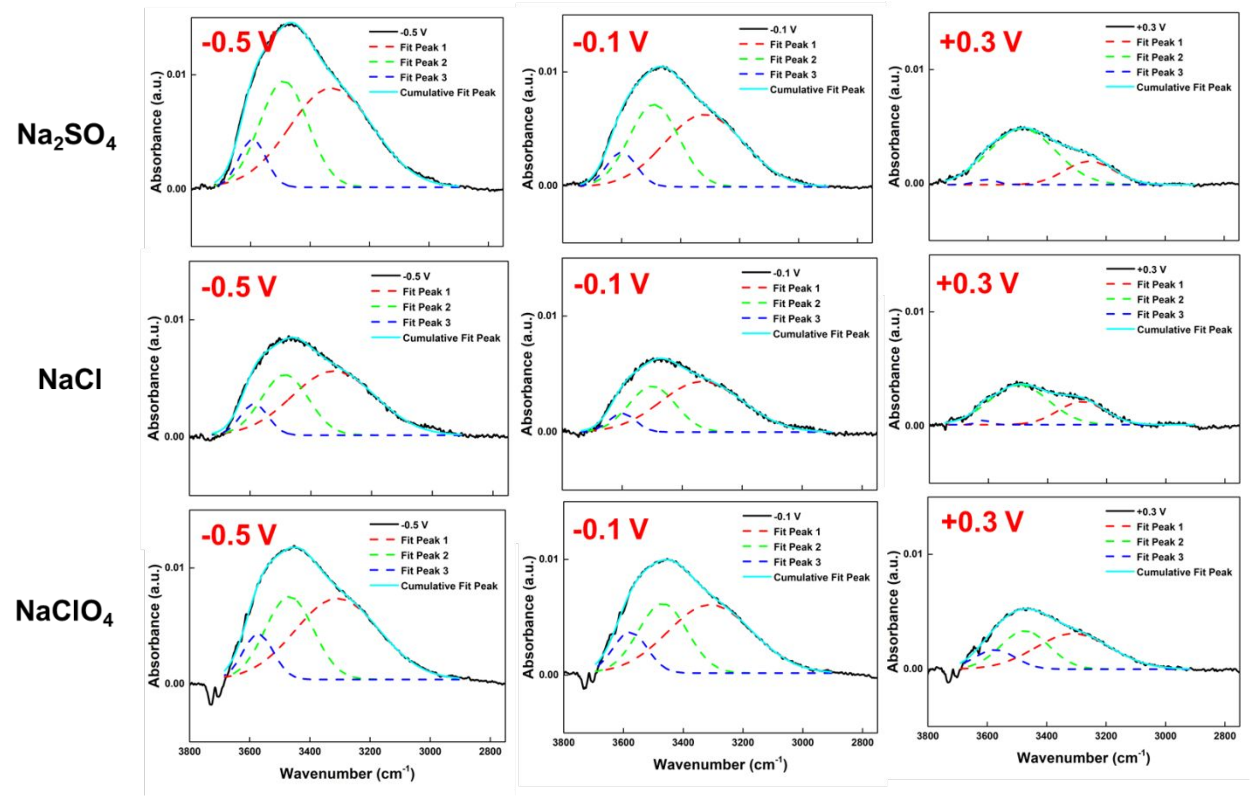

Figure S9. The peak fitting results of PEDOT-PC at OH stretching region in three electrolytes using three Gaussian components: 3295, 3560, and 3590 cm^{-1}, corresponding to FW, IW, and NFW, respectively. –0.5 V, –0.1 V, and +0.3 V are chosen for three potentials to present the distribution change of the water in different electrolytes.

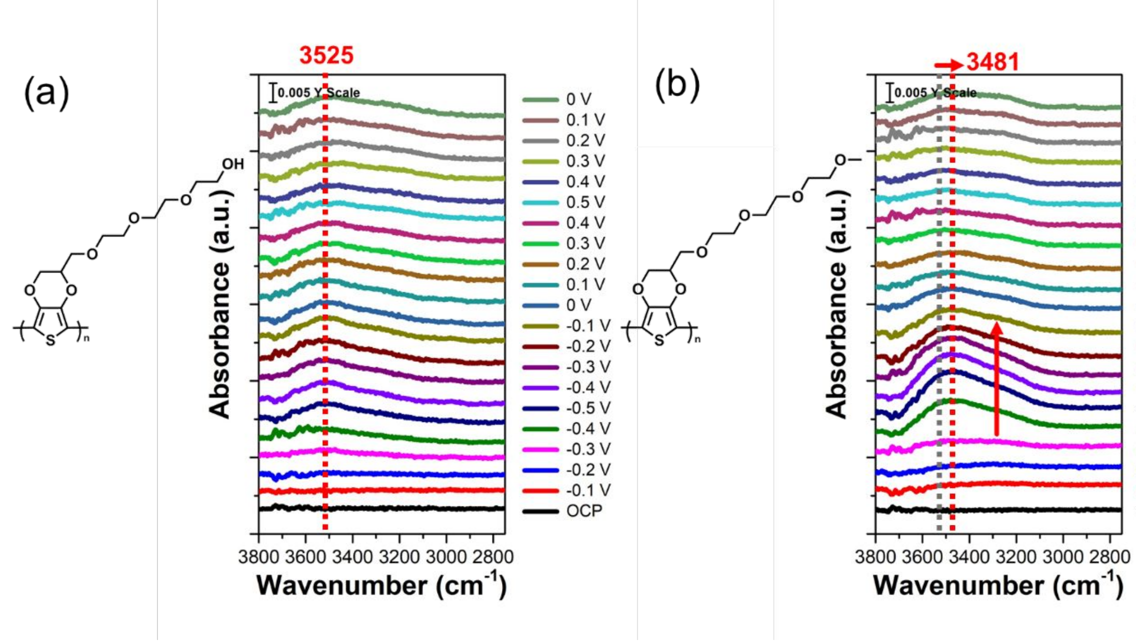

Figure S10. The series of *in-situ* FTIR spectra of (a) PEDOT-EG$_3$OH and (b) PEDOT-EG$_3$OMe in 100 mM NaClO$_4$ at each potential. The applied potential was swept from OCP to –0.5 V, –0.5 V to +0.5 V, and then back to 0 V (vs. Ag/AgCl).

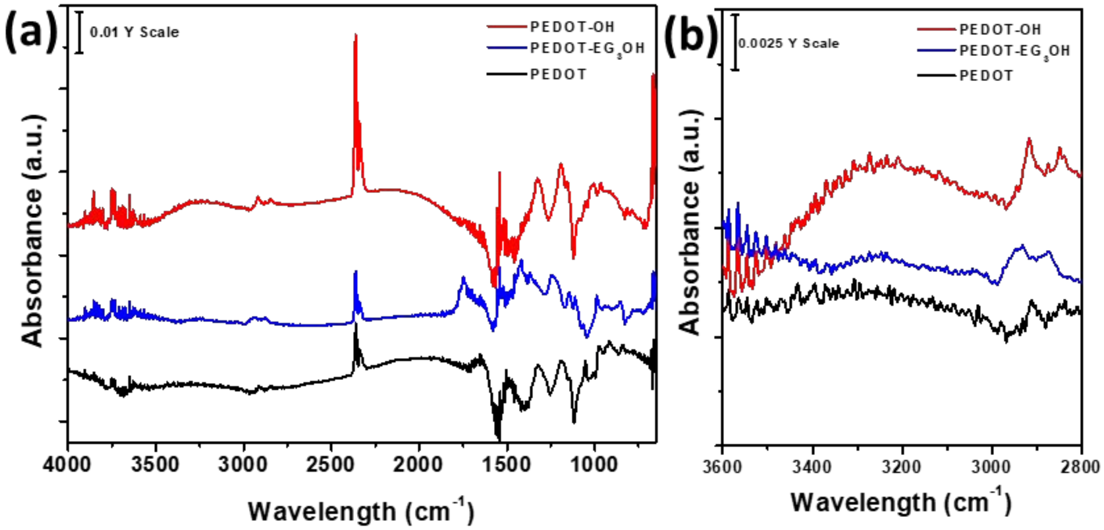

Figure S11. (a) ATR-FTIR spectra of the PEDOT, PEDOT-OH, and PEDOT-EG_3OH films before aqueous media exposure. (b) The corresponding ν (O–H) modes of dry PEDOT, PEDOT-OH, and PEDOT-EG_3OH films.

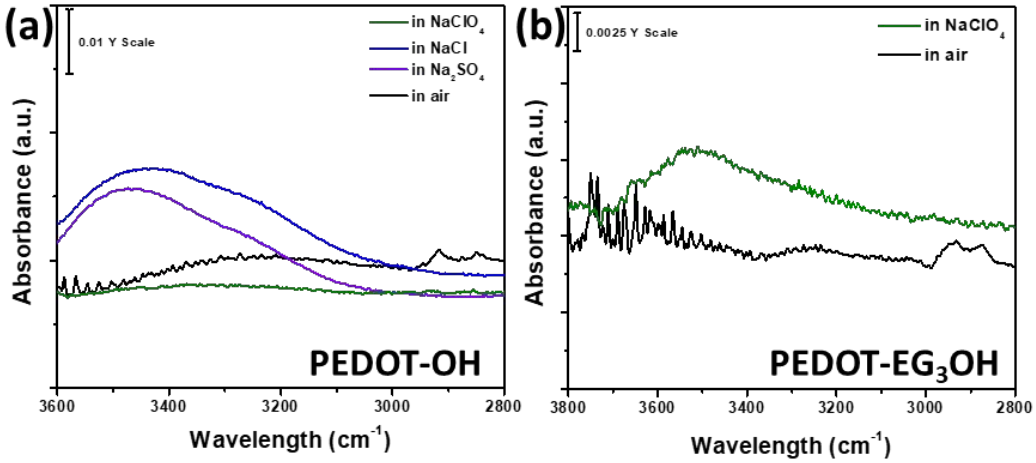

Figure S12. (a) Comparison of O-H stretching modes of dry PEDOT-OH film in air and ν (O–H) modes of PEDOT-OH films in Na_2SO_4, NaCl, and $NaClO_4$ at -0.5 V. (b) Comparison of the ν (O–H) modes of PEDOT-EG_3OH films in $NaClO_4$ solution and in air.

References

[1] a) Y. Chen, Y.-H. Zhang, L.-J. Zhao, Phys. Chem. Chem. Phys. 2004, 6 (3); b) K.-i. Ataka, M. Osawa, Langmuir 1998, 14 (4), 951; c) K.-i. Ataka, T. Yotsuyanagi, M. Osawa, J. Phys. Chem. 1996, 100 (25), 10664.
